# Dynamic light sheet generation and fluorescence imaging behind turbid media

**DOI:** 10.1186/s41476-018-0074-z

**Published:** 2018-02-21

**Authors:** Jale Schneider, Christof M. Aegerter

**Affiliations:** Physik-Institut, University of Zurich, Winterthurerstrasse 190, 8057 Zurich, Switzerland

**Keywords:** Imaging through turbid media, Wave-front shaping, Phase modulation, Light sheet microscopy

## Abstract

**Background:**

Light sheet microscopy became a popular tool allowing fast imaging with reduced out of focus light. However, when light penetrates turbid media such as biological tissues, multiple scattering scrambles the illumination into a speckle pattern and severely challenges conventional fluorescence imaging with focused light or with a light sheet. In this article, we present generation of light sheet type illumination patterns despite scattering.

**Methods:**

We optimize the wave-front of the incoming light to transform the speckle pattern behind the scattering layer into a light sheet within the region of interest. We utilize a fast spatial light modulator for phase modulation and a genetic optimization algorithm. The light pattern behind the scattering layer is detected via a clear detection path and acts as a feedback signal for the algorithm.

**Results:**

We enabled homogenous light sheet illumination behind turbid media and enhanced the signal of fluorescent beads selectively at the desired focal plane up to eight times on average. The technique is capable to compensate the dynamic changes of the speckle pattern as well, as shown on samples consisting of living drosophila pupae.

**Conclusion:**

Our technique shows that not only single foci, but also a homogenous light sheet illumination can directly be created and maintained behind static and dynamic scattering media. To make the technique suitable for common biological settings, where the detection path is turbid as well, a fluorescent probe can be used to provide the feedback signal.

**Electronic supplementary material:**

The online version of this article (10.1186/s41476-018-0074-z) contains supplementary material, which is available to authorized users.

## Background

Scattering of light severely compromises the image quality when turbid media such as thick tissues are observed using conventional fluorescence microscopes. On the one hand, multiple scattering leads to a randomization of the illumination into a speckle pattern; on the other hand, the emitted fluorescence signal gets scrambled as well and cannot be traced back to its origin. Slicing, peeling, clearing etc. hence belong to common tasks of biologists who try to reduce the turbidity of their sample in order to unravel the happenings in tissues and developing animals.

Great technical developments in terms of multi-photon microscopy [1], adaptive optics [2–4], wave-front shaping [5–15], speckle(auto)correlation [16–18], time-reversal [19] and optical phase conjugation [20–28] have improved microscopy in and/or behind turbid media to a great extent. However, the image quality, imaging speed and modalities are still subject to possible improvements.

In this paper, we introduce direct and dynamic formation of variants of light sheet illumination behind scattering layers. Light sheet microscopy [29–31] combines the speed advantage of wide-field imaging with selective plane excitation to reduce out of focus fluorescence and has become a popular tool for biologists for fast three dimensional imaging. Light sheet microscopes illuminate only a thin slice of the sample and the emitted fluorescence from this plane is collected with a detection objective placed perpendicular to the excitation. Nevertheless, traveling through scattering media damages this type of illumination pattern as well leading to a progressive widening of the illumination slice.

We use optical feedback based wave-front shaping to transform the speckle pattern behind a scattering layer into a light sheet within the region of interest. Hence, the setup employs three objective lenses: one objective lens for illumination; a second one facing the first on the opposite side of the sample to provide feedback signal for the optimization; and a third one placed perpendicular to the other two in order to detect the fluorescence signal coming from the excited slice of a fluorescently labelled sample.

**Fig. 1:**
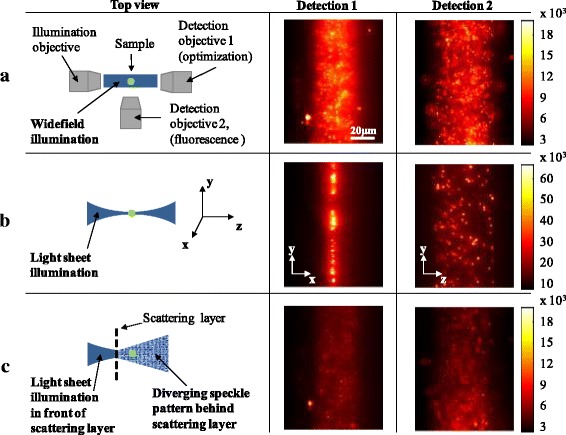
Rows a, b and c of the first column depict different illumination patterns exciting yellow green fluorescent particles in a glass capillary. Detection objectives 1 and 2 capture two orthogonal scenes of the sample as shown in columns 2 and 3. The color scales are in arbitrary units. **a** Wide-field excitation results in images blurred by out of focus signal. **b** Light sheet illumination excites a thin sheet in y–z plane. Detection objective 2 captures a sharp image with negligible out of focus contribution. Detection objective 1 captures a line shaped image. Most of the signal comes from the thinnest part of the sheet with highest intensity. **c** A scattering layer scrambles the illumination into a speckle pattern regardless of its original shape. The resulting images again suffer from out of focus blurring. Note, that detection objective 1 is mainly responsible for providing feedback signal for the optimization to recover a light sheet type illumination behind scattering layer. Nevertheless, it can be used for fluorescence detection as well

Figure 1 explains the setup as well as the problems faced for light sheet microscopy in turbid media in more detail. If one illuminates a sample (in our case a glass capillary filled with fluorescent beads) with a wide-field scheme, both detection objectives capture two perpendicular perspectives of the same scene at their respective focal planes. In this mode, the bright beads at the focal plane are heavily blurred with out of focus fluorescence. If the illumination is shaped into a light sheet, only a thin plane perpendicular to the optical axis of the detection objective 2 is excited. Hence, the detection objective 2 captures an image of clearly distinguishable beads from the sheet with significantly less out of focus signal. Detection objective 1 in that case faces a projection of the light sheet. If its focal plane is set in the middle of the sheet, a thin line of strongly illuminated beads is captured. The length of the line corresponds to the height of the light sheet and its width corresponds to the thickness of the light sheet at the focal position. However, if a scattering layer is placed within the illumination path, the illumination light forms a diverging speckle pattern. The images captured by the detection objectives again become blurry due to out of focus fluorescence and the intensity of single beads depends on their overlap with the illuminating speckles.

The aim of our work is to optimize the spatial phase distribution of the illumination to achieve constructive interference of speckle fields behind the scattering layer and make them form a light sheet within a certain region. With our technique, we recover homogeneous selective plane excitation in spite of the scattering layer.

## Methods

### Optical setup

The most essential part of the setup is a spatial light modulator (SLM, P512-0532 ODP, pixel size of 15 μm, Meadowlark Optics, USA). It spatially modulates the phase distribution of the expanded coherent laser light (488 nm, Spectra-Physics, USA). The “overdrive” feature reduces its response time down to a few milliseconds. The objective lens (HCX PL APO L 20×/0.50, Leica Microsystems, Germany) images the SLM plane onto the surface of the scattering layer with a demagnification of 15× resulting in an illuminated area of approximately 400 μm diameter. The detection objective 1 (HCX PL APO L 40×/0.80, Leica Microsystems, Germany) images the region of interest behind the scattering layer onto the camera (ORCA-Flash 4.0 LT, Hamamatsu, Japan). The magnification is 20×. The detection objective 2 (HCX PL APO L 40×/0.80, Leica Microsystems, Germany) images the light sheet illumination plane onto the same camera with a magnification of 40×. One can switch between the images coming from the two detection objectives via a removable mirror. During fluorescence detection a bandpass filter (FF01-525/45, Semrock Inc., USA) and a notch filter (NF488-15, Thorlabs Inc., USA) are placed in front of the camera.

**Fig. 2:**
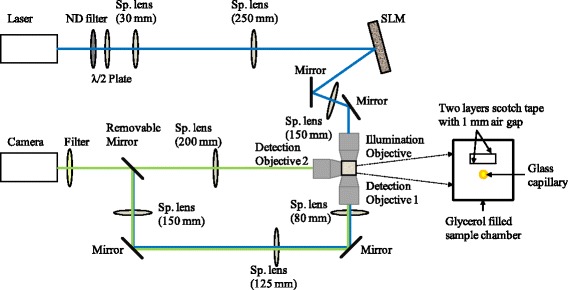
Schematic of the optical setup. ND filter: neutral density filter, Sp. lens: spherical lens, SLM: spatial light modulator. The blue lines represent the excitation; the green lines represent the fluorescence emission. Removable mirror allows switching between imaging arms via detection objective 1 and 2. The filter is also removable to allow for direct imaging of the illumination light. The sample chamber between the illumination objective and detection objective 1 is drawn in more detail on the right side of the schematic showing the position of the scattering layer and of the glass capillary containing the sample

Figure 2 depicts the schematic of the optical setup. When we used a glass capillary as sample, we filled the sample chamber with glycerol to avoid refractive index mismatch on the glass surface which otherwise adds aberrations to the speckle spots.

### Optimization algorithm

Our software is based on genetic algorithms implemented with the optimization app of Matlab® R2015a (The MathWorks Inc., USA). A genetic algorithm solves optimization problems via a natural selection process that mimics biological evolution [32]. Initially, a random population of possible solutions is created. Individuals of the population are then ranked depending on their performance. Good performing individuals become “parents” creating “children” obeying used defined options. Over successive generations, the population evolves to an optimal solution.

**Fig. 3:**
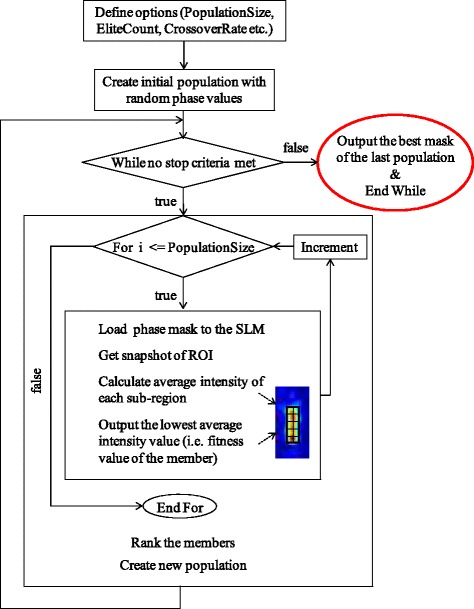
Flow chart of the algorithm. Initially, 20 random phase distributions are applied to the SLM. Every phase mask results in a different speckle pattern at the line shaped region of interest which is images on the camera. The algorithm evaluates the camera images. For this purpose, the ROI is divided into sub-regions as depicted in the inset. The average intensity value of the darkest sub-region of each member serves as its fitness value. The phase masks are then ranked according to their fitness values. A new population is created where the better performing masks contribute more to the phase patterns of the children. To chose the weakest sub-region as figure of merit ensures that not only the overall intensity but also the contribution of the darker parts are optimized. The algorithm can be stopped manually or it stops if the user defined maximum number of generations is created

Our algorithm initially defines a population of 20 spatial phase-mask distributions containing random phase values between 0 and 2π (corresponds to *PopulationSize* of 20 in the terminology of Matlab Inc.). The size of the phase-mask matrix was determined by the segment size grouping single SLM pixels. In our setup, we chose a segment size of 15 × 15 pixels resulting in 34 × 34 matrix of 1156 segments by our 512 × 512 pixels device. In theory, dividing the SLM into more segments containing fewer pixels would lead to a better optimization due to the increased number of freedom degrees [6]. However, crosstalk between the pixels compromises a sharp distribution of different phase values on neighboring pixels. Our preliminary experiments without any scattering media showed that a segment size smaller than 10 × 10 pixels results in unreliable intensity enhancements differing from our simulations. To make sure that every phase distribution is physically correctly set on the SLM, we set the segment size to 15 × 15 pixels. The driver circuit subsequently loads these masks onto the SLM. Every phase distribution results in a different speckle pattern at the region of interest located at the focal plane of detection objective 1. Snapshots of camera images of these speckle patterns for each population member are then evaluated. The goal is to directly achieve a line shaped region of homogenous maximum intensity. As stated in the Background section, light sheet illumination perpendicular to objective lens 2 corresponds to a line shaped intensity projection at the focal plane of objective lens 1. To ensure homogeneity, the line area in the snapshots is divided into sub-regions obeying the Nyquist criteria. In other words, the size of each sub-region is set to be half of the average size of speckles. The algorithm calculates the average intensity of the weakest sub-region of each population member and ranks them. The members providing the highest intensity at their weakest sub-region are ranked higher. The ranking position corresponds to the *expectation value* of the phase mask to be a solution. The best two individuals directly become members of the next generation (called “*EliteCount* of 2” in the terminology of MathWorks, Inc.). Nine other members of the following population (*Children*) are created by crossover (*CrossOver Fraction* of 0.5). For this purpose, 9 *Parent Pairs* from the former generation are selected using *stochastic uniform selection* [32]. This type of selection ensures that the phase masks with the higher rankings are more likely to become a *Parent* and that they can be chosen more than once as well. Then, nine random binary vectors of size 1 × 1156 are generated. Where the binary vector contains a 1, the corresponding phase value of *Parent1*; where the vector contains a 0, the phase value of *Parent2* is taken. These two patterns are then combined to form a new phase distribution (a *Child*). The rest of the new population (in our case 9 remaining members) is created by *Mutation* using *MutationRate* of 0.01. Therefore, 1% of the phase values of nine stochastically uniform selected *Parents* are randomly replaced by other values. The new population is then applied onto the SLM. Figure 3 summarizes our algorithm as a flow chart.

As the algorithm is iterated, the overall intensity increases resulting in a uniformly intense line. The practically achievable height and thickness of a homogenous light sheet depends on the scattering properties i.e. on the average speckle size of the sample, since the light sheets are as thin as the main speckle size. Dainty showed that the average speckle size is inversely proportional to the illuminated area [33]. In our case; a higher turbidity of the sample led to more multiple scattering events and the diffusively illuminated area towards the back end of the sample became bigger. Therefore, the average speckle size at the image plane decreased leading to thinner light sheets and better axial resolution. But the homogenously illuminated area became smaller.

During optimization, the camera exposure time was set to the lowest possible value of 3.021 ms to reduce the time per iteration cycle. For the *PopulationSize* of 20, each generation took approximately 300 ms including mask loading to the SLM, SLM response time, camera exposure time, signal evaluation, ranking and creation of the next generation. The illumination intensities were low and varied between 0.5 and 1 μW at the back focal plane of the objective lens providing just enough initial signal for an efficient optimization.

## Results

### Light sheet behind scotch tape

**Fig. 4:**
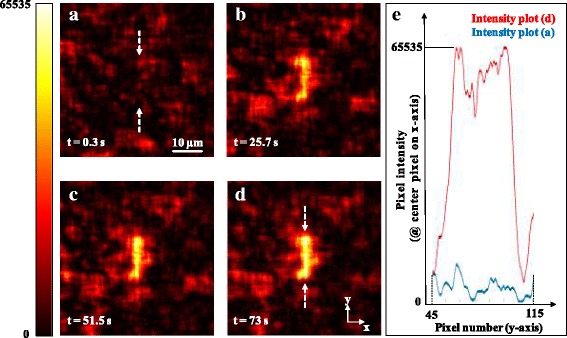
**a** The originally wide-field pattern of the excitation light gets scrambled after traveling through scotch tape. **b**–**d** Changing the phases of different parts of the incoming light provides constructive interference of the scattered light paths. The intensity within the region of interest increases while the algorithm optimizes the spatial phase distribution of the incoming light. The images are taken with detection objective 1 having its focal plane set 1 mm behind the turbid medium. The colorbar in arbitrary units is valid for all the subfigures. **e** Intensity profiles over the line-shaped regions in subfigures (**a**) and (**d**). The arrows indicate the starting and ending points of the line plots. The average intensity within the ROI in (**d**) is ~ 12 times higher than the average intensity in (**a**)


We first performed wave-front shaping of the illumination behind scotch tape as a turbid medium (most samples consisted of two layers of scotch tape with an air gap of 1 mm in-between). The scotch tape mimics biological scenarios where scattering occurs due to refractive index mismatch between the sample and its holder and surrounding medium. It also imitates refractive index variation within the specimen. As depicted in Fig. 4(a), the illumination forms a random speckle pattern. The algorithm then defines an area of 13.5 μm × 2.7 μm as a target region and evaluates the intensity distribution within this area for different spatial phase distributions of incoming light. The snapshots in Fig. 4(b), (c), (d) show the intensity increase while the genetic algorithm iteratively continues creating new populations. The real time video (recording rate: 33 fps, display rate: 30 fps, field of view 52 μm × 39 μm) of the optimization process can be found in Additional file 1: Video S1. On these samples, the intensity within the region of interest (ROI) could be enhanced 12 to 15 times compared to the average background intensity. The algorithm was stopped manually. Optimizing too long led to camera saturation and hindered the evaluation of light sheet homogeneity.

Additional file 1:Video of dynamic optimization algorithm at work, leading to a light sheet behind turbid media. (MP4 9024 kb)


The thicknesses of the light sheets *ω*_0_ at the focal plane varied between 2.7 and 3.4 μm. We measured the thickness using our camera images. The camera pixel size of 6.75 μm and the magnification factor of 20× on the detection arm resulted in a pixel size of 6.75 μm/20 = 0.3375 μm on the image plane. The numerical aperture *N.A.*, which focuses the speckle illumination on one axis forming the light sheet, can then be calculated according to Eqs. (1)–(3):

1 $$ N.A.=n\cdot \mathit{\sin}\;\theta $$

with *θ* being the half opening angle of the focused beam and *n* the refractive index of the medium where the beam travels through. Due to small angle approximation, Eq. (1) can be simplified as:

2 $$ N.A.\sim n\cdot \theta $$

Assuming the Gaussian beam approximation to be valid, Eq. (2) can be rewritten as:

3 $$ N.A.\sim n\cdot \lambda /\left(\pi \cdot {\omega}_0\right) $$

with *λ* being the wavelength of the laser. We filled our sample chamber with glycerol with a refractive index of 1.47. Laser illumination of 488 nm resulted then in a *N.A* of 0.067–0.085. The Rayleigh range of the light sheets varied 47–74 μm according to Eq. (4).

4 $$ {z}_r=\pi \cdot {\omega_0}^2/\lambda $$

with *z*_*r*_ indicating the Rayleigh range.

### Fluorescence excitation with light sheet behind turbid media

**Fig. 5:**
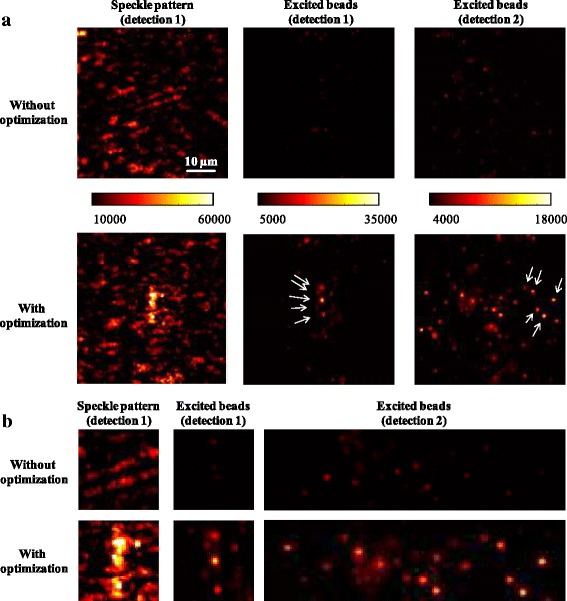
**a** The upper and lower panels compare images before and after optimization. The leftmost column shows the illumination speckle patterns behind scotch tape observed by detection objective 1. The middle and right columns show the fluorescence signal excited with these speckle patterns captured by the detection objectives 1 and 2 respectively. The optimization provides strong enhancement of the fluorescence signal within the region of interest at desired y–z plane. Each image pair per column shares the same color scale in arbitrary units. **b** Zoomed in images corresponding to regions of interest from part (**a**)

Subsequently, we used the created light sheets behind scotch tape to image fluorescent samples. For this purpose, we filled a round capillary with an inner diameter of 50 μm (VitroCom, USA) with a 10:1 dilution of glycerol containing 1 μm diameter yellow-green beads (Invitrogen FluoSpheres®, USA). The capillary was then set 1 mm behind the tape into the focal plane of detection objective 1. Figure 5(a) shows exemplary the speckle patterns before and after optimization as well as the corresponding fluorescence excitation recorded with both detection objectives. Figure 5(b) zooms into the region of interest of the images from part (a). Typical enhancement factors of the excitation intensity within ROI varied on these samples 7–14. The signal of the excited beads was enhanced 4–8 times on most beads.

The data supports the Rayleigh range of 47 μm – 74 μm calculated above. The capillary’s diameter of 50 μm is within this range and the images taken with the detection objective 2 show no significant signal decrease on the edges of the capillary. This would have been the case, if the Rayleigh range were much smaller than the capillary’s diameter (i.e. the intensity of the focused beam on the edges were significantly lower than the half of its center value). We also performed experiments with capillaries of different diameters filled with different concentrations of beads. Our image field of ca. 345 μm × 345 μm on detection path 2 allowed imaging of bigger capillaries easily. In general, bigger capillaries led to higher signal contribution from the out of focus layers. High bead density hindered the localization of the intensity enhanced plane. On the other hand, a low bead density led to a poor representation of the illuminated plane. We concluded, that our current configuration mentioned above allowed the best performance analysis of the light sheet creation.

### Continuous optimization to compensate sample dynamics

**Fig. 6:**
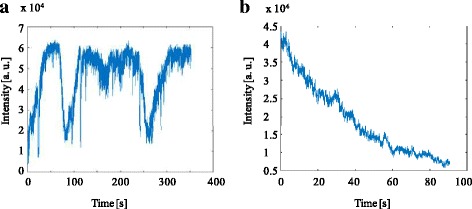
**a** Fluctuation of the average intensity of the light sheet behind a living drosophila pupa (see also Additional file 2: Video S2). It takes a few tens of seconds to recreate a new light sheet, if the speckle pattern changes significantly due to breathing. **b** Decay of the average intensity with time after stopping the algorithm (see also Additional file 3: Video S3). The optimization algorithm needs to run continuously to compensate for small changes in the speckle pattern. Otherwise, the average intensity decays on a time scale of roughly 30 s


The dynamics of living biological samples challenges every type of microscopy and causes motion blur. Here, we face the additional difficulty that the optimization process is susceptible to any changes in the speckle pattern, which easily occur during movement of the sample or diffusion of molecules inside it. Hence, the algorithm needs to run continuously to keep up with slow as well as abrupt changes while optimize as quickly as possible while living samples are observed. To test the limits of our system on dynamic samples, we created light sheets behind living drosophila pupae. We choose pupae at stages P4 – P7 [34]. The earlier stages are dominated by the buoyant behavior of the prepupa containing a moving air-bubble leading to a change in the speckle patterns on a millisecond time scale. In contrast, the later stages offer very slow changes on the scale of minutes to hours. During the selected pupal stages, breathing is considered to be responsible for abrupt changes of the speckle pattern and molecular diffusion for the slow changes in seconds scale. The optimization in general took 30 s – 60 s to create a light sheet. On sudden events, the optimized intensity distribution collapsed but was regenerated within 20 s – 30 s. Additional file 2: Video S2 shows such a light sheet generation behind moving pupae in real time (recording rate: 33 fps, display rate: 30 fps, field of view 52 μm × 39 μm). Figure 6(a) depicts the corresponding drops and recoveries of the average intensity over time. Additionally, Additional file 3: Video S3 (recording rate: 33 fps, display rate: 30 fps, field of view 52 μm × 39 μm) replays the decay of an optimized light sheet, in the absence of an abrupt event, once the algorithm stops running continuously. The corresponding time dependence of the average intensity is shown in Fig. 6(b). As long as the light sheet was stable, the excitation light was enhanced ~ 8 times compared to the average background intensity.

Additional file 2:Fluctuation of the average intensity of the light sheet behind a living drosophila pupa. (MP4 20,129 kb)



Additional file 3:Temporal decay of the average intensity of the light sheet behind a living drosophila pupa after stopping the algorithm. (MP4 14,614 kb)


### Homogeneity of the light sheets

**Fig. 7:**
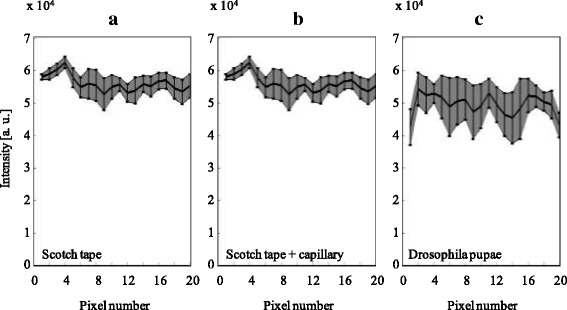
Averaged line plots (black lines) and their standard deviation (shaded area with errorbars) over the light sheets in the middle of the ROI at the focal plane of the detection objective 1 (**a**) for optimizations behind scotch tape (**b**) for optimizations inside the glass capillary behind scotch tape and (**c**) for optimizations behind living drosophila pupae

We evaluated the homogeneity of the light sheets based on intensity line plots from images captured via detection path 1. In Fig. 7, the intensities over 20 pixels in the middle of the ROI are plotted as done in Fig. 4(e). The line plots are averaged over four different measurements, where the shaded area indicates the standard deviation (Single line plots without averaging can be found in Additional file 4: Figure S1). Figure 7(a) depicts the enhancement behind scotch tape, Fig. 7(b) that using a glass capillary behind scotch tape as fluorescent sample and Fig. 7(c) shows intensity distributions after optimizing behind drosophila pupae. The most homogeneous light sheets were obtained after optimizing behind scotch tape only. Here, the maximum deviation of the intensity of a single pixel from the mean value was ~19%. When a capillary was placed behind the scotch tape, the pixels with the largest deviation were ~30% from the mean. Finally, drosophila samples showed intensities deviations of ~35%. However since the light intensity enhancement factor of the light sheet is about eight, this still presents a clear illumination of the region of interest. Moreover, in most cases, a fluorescent structure behind turbid media is not excited by only one pixel but by an ensemble of pixels. Therefore, the effective fluorescence signal can be expected to be more homogenous than the line plots of the excitation light.

## Discussion

Arbitrary intensity distributions rather than a single focus behind turbid media have already been demonstrated. Malavalli et al. created an array of multiple foci behind turbid media by first determining the phase distribution to create a single focus and then adding linearly another phase mask to it [11]. This strategy can be applied to create a light sheet as well. The second mask to be added must then act as a negative cylindrical lens to elongate the focus into a light sheet. However, several disadvantages would come along. First, the region of possible elongation is fundamentally limited by the memory effect (The memory effect describes the wave-front correlation valid within a certain angular range [35, 36]). Additionally, the focal length of the cylindrical lens requires fine-adjustments to match exactly to the position of the focus along the optical axis. Finally, the intensity within the focus will be spread over a larger region and thus necessitates dynamic and/or static intensity adjustments during and after the optimization. Also Vellekoop et al. showed in his pioneering work optimization on multiple spots [6]. However, in both works, no special effort was put to ensure a homogenous intensity distribution like we did as mentioned in Results section.

The enhancement factors of excitation intensity within ROI varied 7 to 15. Instead of creating a light sheet, one could create one single focus with higher enhancement [6] and scan it over the desired area as well [9, 13]. Our setup creates the light sheet directly and requires no scanning system and additional optical components to create conjugate planes. This makes the microscope simple and robust. As long as the signal to background ratio is enough, the lower enhancements compared to the single focus optimization do not hinder the advantages of light sheet imaging.

In our experiments, the excitation beam and the fluorescence signal could reach the camera directly without being scattered. However, in most biological settings, the detection path will not be clear and the signal coming from the sample plane will be aberrated and scattered as well. This would hinder the evaluation of the light sheet using its image on the camera. In this case, this type of optimization using the direct beam can only be performed by weak scattering on the detection side where significant amount of the direct beam reaches the camera as ballistic photons. In such a scenario, adaptive optics tailored for the detection arm of light sheet microscopes can be implemented as well [37]. If the detection side is subject to severe scattering, as we assume for many biological applications, alternative approaches using fluorescent probe as feedback signal can be applied. For this purpose, a bright and isolated fluorescent particle can be placed at the sample plane of interest. A fluorescently labelled structure of the specimen itself might act as a source as well. The scattered fluorescence signal from the source can be focused on a single point detector on the back side of the overall turbid media. The wave-front of the illumination can then be optimized to increase the signal at the detector which corresponds to an intensity increase of the excitation at the region of interest. Such methods using the image brightness as a metric for different optimization algorithms have already been used for implementation of adaptive optics in confocal and multiphoton microscopy as compared by Wright et al. [38]. To use a labelled structure in the specimen is surely the better choice than invasively bringing a feedback source into the sample which can also destroy the structures in the ROI. However, the labelling might be then too dense leading to a mixed signal from a bigger area. On the other side, sparse labeling can bleach quickly. In that case, quantum dots would offer practically non-bleaching signal. The proposed method would work for creating a single focus in the sample which can be then scanned within the range of memory effect. To collect signal from a line shaped source instead of a point source can lead to an increase of overall intensity of a light sheet. However, the homogeneity cannot be deduced from the signal increase at the detector since the scattered fluorescence from different parts of the line shaped source would be mixed up. To still ensure homogeneity and create directly a light sheet without scanning, a chain of spectrally different quantum dots can act as feedback source. Many quantum dots can be excited by the same wavelength but will emit fluorescence at a different wavelength. Optimizing on different colors from adjacent spots can lead to a more homogenous intensity increase of light sheet type illumination.

Our optimization speed in the range of tens of seconds is moderate. Cui demonstrated focusing through highly scattering media within 400 ms via spatial frequency modulation [39]. Tang et al. used nonlinearity assisted iterative optimization (IMPACT) and focused light through mouse brain tissue and skull within ~5 s [15]. In a later in vivo study on neurons, the optimization time was reduced down to ~2 s [40]. Liu et al. carried the time scale of time-reversed ultrasonically encoded (TRUE) optical focusing down to a few milliseconds [23]. Wang et al. achieved similar speeds via digital optical phase conjugation employing a digital micro-mirror device and a FPGA with custom firmware [27]. Even focusing behind turbid media in sub-millisecond time scale was shown by the process of field self-organization inside a multimode laser cavity [41]. All these works offer higher speeds than our system. However, they require elaborate optical paths and/or dedicated electronic hardware and software which makes the systems complex and expensive. The ones utilizing phase conjugate mirrors show also wavelength dependency. Once established, our setup is easy to adjust, can tolerate misalignments and is inexpensive. The calibration of the SLM can easily be scaled for different wavelengths. This makes our system suitable for usage not only in physics labs but also in biology labs. Another faster and simple approach was developed by Edrei et al. based on so called shower curtain effect [42]. But it requires coherent light which makes fluorescence light unsuitable as contrast mechanism for imaging. A moderate optimization speed was also achieved by N’Gom et al. [43]. Here, portions of the transmission matrix of the scattering medium are constructed by computationally intensive semi-definite programming. Single and multiple foci were created behind turbid samples with a speckle persistence time in minutes scale. Besides, imaging inside turbid media has been enabled by other computational methods utilizing compressive sampling. Duran and Tajahuerce et al. used transmitted light captured by a single pixel camera and could reconstruct sparse high contrast objects hidden in turbid media [44, 45]. However, imaging of fluorescence emitting low contrast objects still remained a challenge.

## Conclusion

We have shown that our wave-front shaping microscope enables homogenous light sheet illumination of fluorescent samples behind turbid media. This type of illumination is particularly advantageous while exciting a big area at once with reduced out of focus light. Our setup can compensate sample dynamics on a time scale below minutes and suits for example applications for developmental biology such as organ growth and morphogenesis in embryo and larvae. Another possible application area is optical coherence tomography (OCT) in ophthalmology where the backscattered light from retina within rather “clean” eye can serve as feedback signal. Dedicated wave-front correction techniques improving the penetration depth and image quality in OCT have already been shown by Fiolka et al. [46] and Yu et al. [47]. These works can be extended to achieve light sheet type illumination as well.

This work of illumination optimization presents a necessary first step for imaging of turbid biological scenes, which eventually needs to be complemented with methods to recover the fluorescent image after scattering. These complementary methods need to be performed based on the signal from detection path 2 facing the light sheet plane, since phase aberrations would occur differently on our two different detection sides.

## Additional files

Additional file 4: Raw data used for averaging in Fig. 7. (DOCX 98 kb)

## Abbreviations

IMPACT: Iterative multiphoton adaptive compensation technique
ND filter: Neutral density filter
SLM: Spatial light modulator
TRUE: Time-reversed ultrasonically encoded
