# Supplementary material for: Dynamic light sheet generation and fluorescence imaging behind turbid media
Source: J Eur Opt Soc Rapid Publ. 2018 Feb 21;14(1):7. doi: 10.1186/s41476-018-0074-z (PMC5846999; doi:10.1186/s41476-018-0074-z)
Supplement: Supplementary file 1 [file 41476_2018_74_MOESM4_ESM.docx]

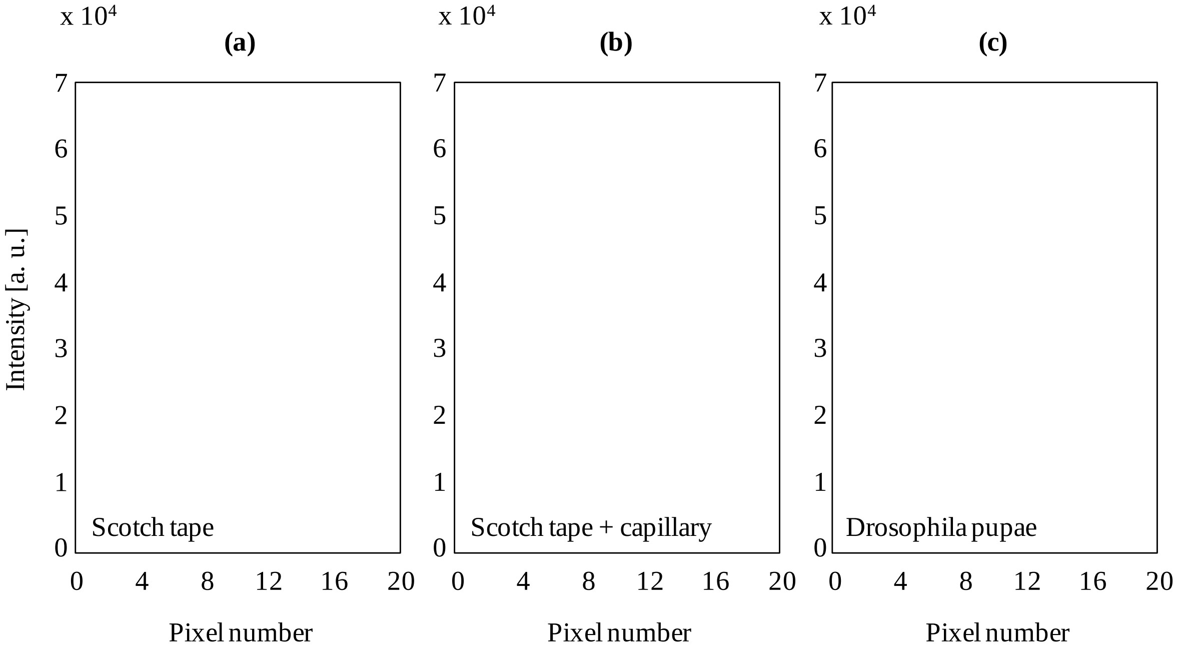
x 10^4
(a)
(b)
(c)
Intensity [a. u.]
7
6
5
4
3
2
1
0
0
4
8
12
16
20
Scotch tape
Scotch tape + capillary
Drosophila pupae
Pixel number
